# Risk of death for hematological malignancies for residents close to an Italian petrochemical refinery: a population-based case-control study

**DOI:** 10.1007/s10552-014-0468-1

**Published:** 2014-10-05

**Authors:** Andrea Micheli, Elisabetta Meneghini, Mauro Mariottini, Marco Baldini, Paolo Baili, Francesca Di Salvo, Milena Sant

**Affiliations:** 1 Epidemiologia Analitica e Impatto Sanitario, Fondazione IRCCS Istituto Nazionale dei Tumori, Via Venezian 1, 20133 Milan, Italy; 2 Direzione Scientifica, Fondazione IRCCS Istituto Nazionale dei Tumori, Via Venezian 1, 20133 Milan, Italy; 3 Osservatorio Epidemiologico Ambientale Regione Marche, ARPAM, Servizio Epidemiologia Ambientale, Via C. Colombo 106, 60127 Ancona, Italy

**Keywords:** Hematological malignancies, Residential exposure, Petrochemical refinery

## Abstract

**Purpose:**

We investigated the risk of death for hematological malignancies (HMs) in the area surrounding an Italian petrochemical refinery, where atmospheric concentrations of benzene (known carcinogen) had not been adequately monitored in the past.

**Methods:**

We performed a population-based case-control study, using conditional logistic regression to estimate odds ratios (ORs) of HM death, with 95 % confidence intervals (CIs), and *p* trends, in relation to tertiles of time-weighted average residential proximity to the refinery. We identified 177 HM deaths and 349 sex- and age-matched controls from municipal files. Confounding factors were investigated from interviews with consenting relatives for 109 HM deaths and 178 matched controls.

**Results:**

For males and females combined, risk of HM death was unrelated to residential proximity. For females, ORs of HM death by increasing tertiles of proximity were 1, 2.74 (95 % CI 1.48–5.09, significant) and 1.49 (95 % CI 0.76–2.92) (*p* trend 0.184). For the subgroup of persons who plausibly spent most of their time at home (long-term retired, homemakers or unemployed, 53 cases, 79 controls), the ORs of leukemia plus non-Hodgkin lymphoma death (38 cases, 56 controls) by increasing tertiles of proximity were 1, 3.44 (95 % CI 1.04–11.37, significant) and 3.25 (95 % CI 0.82–12.87) (*p* trend 0.083).

**Conclusions:**

No increased risk of HM death for males and females combined living close to the refinery was found. However, the findings for females and a subgroup plausibly spending most of their time at home suggest a relation between increased risk of HM death and residential proximity to the refinery.

## Introduction

Hematological malignancies (HMs) are a heterogeneous group of diseases often classified, for public health and epidemiological purposes, into Hodgkin lymphoma, non-Hodgkin lymphoma, multiple myeloma, and leukemia—entities that account for 6–8, 40–44, 15–16, and 34–36 %, respectively, of incident HMs in developed countries [1]. Although recent changes in the classification of these diseases make it difficult to compare past and recent trends, the incidence of non-Hodgkin lymphoma and, to a lesser extent, of multiple myeloma seems to be increasing [2–4]. Epidemiological studies have not identified risk factors that plausibly explain the incidence increase, or geographic variations in incidence, even though progress in immunology and molecular biology has increased understanding of pathogenetic factors for these diseases [2–5].

Risk factors for leukemia and non-Hodgkin lymphoma include family history, particularly for lymphoma [3], genetic propensity [2, 3, 6], infectious agents (notably human T cell leukemia virus-1, human immunodeficiency virus-1, hepatitis C virus, and Epstein-Barr virus) [2, 3], smoking [2, 6], chemotherapy [2, 6], ionizing radiation (including radiotherapy) [2], low-frequency magnetic fields (childhood leukemia) [7], and occupational exposure to chemicals (herbicides, pesticides, formaldehyde, rubber industry chemicals, and benzene) [2, 3, 8]. Benzene was included in the International Agency for Cancer Research (IARC) list of substances carcinogenic to humans in 1982 after the accumulation of abundant documentation that it is a cause of some forms of leukemia (acute myeloid leukemia, acute non-lymphocytic leukemia) [9]. More recently, benzene has also been related, with more limited evidence, to acute lymphocytic leukemia, chronic lymphocytic leukemia, non-Hodgkin lymphomas, and multiple myeloma [8].

Although benzene is a ubiquitous contaminant (it is present in petrol) and there is abundant information attesting to its carcinogenicity, public awareness of the hazards of exposure to benzene tends to be low. Petrochemical refineries and other petroleum facilities are well-known sources of benzene [8].

Numerous studies have analyzed HM incidence or HM mortality in areas around petrochemical plants [10–18]. Significantly increased incidence [11, 13] and mortality [12] of leukemia; non-Hodgkin lymphoma incidence [15] and mortality [18]; and HM mortality [17] have been found. However, two studies found no significant increases in risk [10, 16], while another reported decreased risks of HM among persons living close to petrochemical plants [14]. Some of these studies [13, 14] found low atmospheric concentrations of benzene close to the plants investigated.

In this paper, we present the results of an Italian population-based case-control study to evaluate the risk of HM-related death among people living close to the petrochemical refinery of Falconara Marittima (Falconara), Ancona Province, Region of the Marches, Italy.

## Materials and methods

### Background

The refinery, located close to a residential area in the Municipality of Falconara, is at high risk of being a pollution source [19]. Atmospheric levels of the pollutants ozone, sulfur dioxide, hydrogen sulfide, nitrogen dioxide, benzene, and total non-methane hydrocarbons (NMHCs) have been monitored around the refinery since 2005 [20]. Average annual atmospheric concentrations of benzene and total NMHCs, archived by Province of Ancona authorities, have usually been reported as within the limits prescribed by Italian law, even though these limits have reduced over time (10 µg/m^3^ in 2005, 5 µg/m^3^ in 2010) [21].

In the early years of the new millennium, residents, local doctors, and oncologists expressed renewed concerns that the incidence of leukemia and other HMs might be increasing in the area and requested an investigation into cancer risks. A study funded by the Region of the Marches in 2003 found that mortality from leukemia and other HMs was increasing (nonsignificantly) over time in the municipality of Falconara and adjacent municipality of Chiaravalle, in the context of decreasing or stable HM mortality in the Province of Ancona and Region of the Marches from 1984 to 2000 [22].

However, because monitoring data had shown that known carcinogens were present in the atmosphere, with a support of the Region of the Marches, a further study was initiated to investigate the risk of death from leukemia, non-Hodgkin lymphoma, and other HMs [23–25] (an incidence study was difficult if not impossible as the area was not covered by a cancer registry). The results of this study are presented here. A detailed report of this study’s findings has also been delivered to the Region of the Marches; findings have also been presented at public meetings in the area around the refinery.

### Cases and controls

**Fig. 1:**
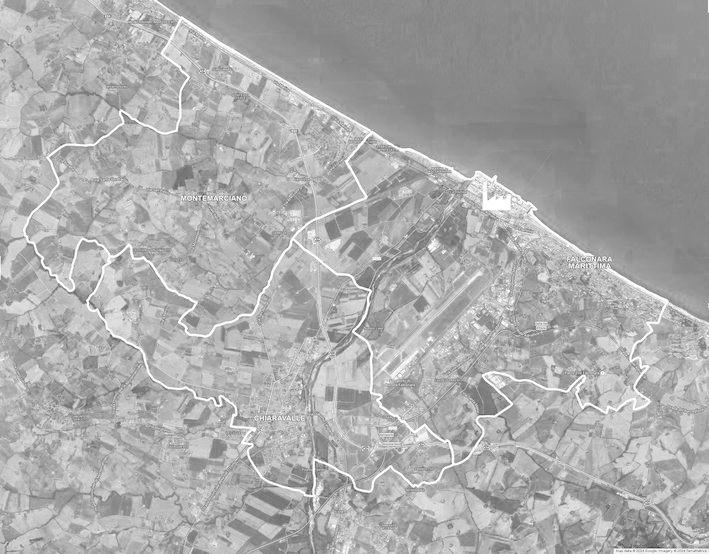
Map showing refinery and neighboring municipalities of Falconara Marittima, Chiaravalle, and Montemarciano. Municipality boundaries and the refinery centroid are indicated in *white*. The dark area to the north-east is the Adriatic Sea

Following results of the previous study on HMs mortality [22] (see above), the study area comprised the urban municipalities of Falconara and Chiaravalle. In order to enlarge the study base, the adjacent municipality of Montemarciano was also included. The study area was approximately 65 km^2^ (Fig. 1) . In 2003, the population of these municipalities was 54,994, of whom 51.5 % were female. HM-related deaths occurring between 1 January 1994 and 31 December 2003 (study period) were identified from death certificates made available by the Italian National Institute of Statistics (ISTAT). The death certificate had to specify HM as the immediate or underlying cause of death. A total of 177 HM-related deaths (89 males and 88 females) were identified. Two controls were selected at random from the risk set (eligible controls) for each case using the risk set sampling method [26]. In accord with this method, the risk set for each case consisted of study area residents alive at the date of case death (index date) and with the same sex and age (± 2.5 years for cases <85 years; for cases ≥85 years (19 % of total), the risk set was all residents ≥85 years). Controls were matched to cases by sex, and age, and by being alive at the index date. The risk set sampling method includes the possibility that persons can be chosen as controls more than once for different cases (in fact, four persons were extracted twice as controls), and persons can be controls at a given index date even if they later become cases (two controls became cases).

Residences were ascertained by the consultation of municipal records. We were able to obtain a complete history of official residencies for all 177 cases, and for 349 matched controls (all except five, so that five sets had one case and one control) (Table 1). Potential confounding factors were investigated by interviewing relatives of 109 (62 % of 177) cases and 186 (53 % of 349) matched controls who consented to be interviewed. The analyses were performed on anonymous data. Study design and procedures are described in a report formally approved by the Region of the Marches [27].

**Table 1:** Characteristics of study participants

	All study participants				Participants with relatives interviewed			
	Cases		Controls		Cases		Controls	
	No.	%	No.	%	No.	%	No.	%
HM (ICD-9 code)^a^								
Leukemia (204–208)	79	44.4	–	–	51	46.8	–	–
Non-Hodgkin lymphoma (200, 202)	56	31.6	–	–	34	31.2	–	–
Myeloma (203)	39	22.0	–	–	22	20.2	–	–
Hodgkin’s disease (201)	3	1.7	–	–	2	1.8	–	–
Sex								
Male	89	50.3	176	50.4	57	52.3	97	52.2
Female	88	49.7	173	49.6	52	47.7	89	47.8
Age (years)								
Males								
0–24	3	3.4	6	3.4	3	5.2	5	5.2
25–74	45	50.5	90	51.1	27	47.4	46	47.4
≥75	41	46.1	80	45.5	27	47.4	46	47.4
Females								
0–24	1	1.1	2	1.1	0	0.0	0	0.0
25–74	31	35.2	62	35.8	16	30.8	24	27.0
≥75	56	63.6	109	63.1	36	69.2	65	73.0
Retired, homemaker, or unemployed^b^								
<10 years	–	–	–	–	49	45.8	73	39.5
≥10 years	–	–	–	–	58	54.2	112	60.5
Totals	177	100.0	349	100.0	109	100.0	186	100.0

^a^ICD-9 code: codes of International Classification of Diseases, ninth revision [35]

^b^Three participants (two cases and one control) had missing information on occupational status

### Exposure

As proxy of cumulative exposure to the refinery pollutants, we calculated the time-weighted average residential proximity for each study participant. Individual residential proximities (*µ*_i_) were defined as the reciprocal of the distance in km (*d*_i_) of the residence from the refinery (*µ*_i_ = 1/*d*_i_) for residences within the study area. Each proximity (*µ*_i_) was weighted by the time (*t*_i_) spent in each residence. Thus, the time-weighted average residential proximity is given by the following:

$$\frac{{\sum\limits_{\text{i}} {\mu_{\text{i}} \cdot t_{\text{i}} } }}{{\sum\limits_{\text{i}} {t_{\text{i}} } }}.$$

Only times pertaining to residences in a 15-year interval (time window) were considered. Participants who resided outside the study area for some part of the time window had zero proximity for that part of the window. To define this 15-year window, we excluded residences preceding the index date by over 20 years to take account of data indicating that the latency between exposure and cancer mortality in workers exposed to benzene is of this order of magnitude [28–30]. We also excluded the 5 years prior to death for persons ≥25 years (or 2 years prior to cancer death for persons under 25 years, four cases), to account for survival between the unknown diagnosis date and the date of death.

The centroid of the refinery area was defined using the GIS MapInfo Professional software (release 8.0). The geographic coordinates of residences were measured individually using a global positioning device (Garmin GPSMAPS 60CS). Distances of residences to the refinery centroid were then calculated. The distances of residences—and time spent in residences—to the nearest power line were also determined in order to consider the confounding effect of extremely low-frequency magnetic fields.

We performed ancillary analyses in which we considered as exposure proxies (a) the distance of residence lived in for longest time during the time window to the refinery centroid (main residence) and (b) the distance of the most recent residence, inhabited for at least a year, within the time window (most recent residence).

### Interviews

Trained interviewers blind to case/control status carried out face-to-face interviews with a relative (sometimes two), who could be spouse, child, sibling, parent, nephew/niece, or brother/sister-in-law of the case/control. Interviewers used an ad hoc questionnaire developed from a preexisting questionnaire [31] and adapted them to the requirements of the present study in consultation with epidemiologists. A copy of the questionnaire was sent out in advance to all those interviewed. Interviewee addresses were acquired from municipal databases.

Relatives of 531 participants were sought initially, but relatives of 38 (12 cases, 26 controls) were not traced, so relatives of 493 participants were contacted: 346 of these (pertaining to 95 cases and 251 controls) agreed to be interviewed at initial contact. Relatives of an additional 14 cases and 16 controls agreed after having been contacted by family doctors. Questionnaires were obtained for relatives of 109 cases and 186 matched controls (total 295 participants, 141 females). The questionnaire solicited information on active and passive smoking; marital status; education; family history of HM; history of congenital or viral conditions potentially predisposing to HM; occupational history from age 15 (two approaches, see below); residences actually inhabited prior to index date (available for 106 cases and 178 matched controls); and potential HM risks related to the characteristics and environment of habitations (Table 2).

**Table 2:** Characteristics of 295 participants (109 cases and 186 matched controls) whose relatives were interviewed

Participant characteristics and confounding factors		Cases		Controls	
		No.	%	No.	%
Education: at least eight years of schooling	No	89	82.4	158	84.9
	Yes	19	17.6	28	15.1
Marital status	Not married^a^	51	47.2	84	45.2
	Married	57	52.8	102	54.8
Smoking^b^	Never smoked	57	53.8	106	57.0
	Former smoker	32	30.2	46	24.7
	Current smoker	17	16.0	34	18.3
First degree relative with HM^c^	No	84	87.5	164	97.6
	Yes	12	12.5	4	2.4
Disease associated with increased HM risk^d^	No	105	97.2	184	98.9
	Yes	3	2.8	2	1.1
Occupational exposure (OCCAM matrix)^e^	<10 years	97	90.7	169	91.8
	≥10 years	10	9.3	15	8.2
Occupational exposure (MATline matrix)^f^	<10 years	98	91.6	173	94.0
	≥10 years	9	8.4	11	6.0
Resident in urban area^g^	<10 years	10	9.4	25	14.0
	≥10 years	96	90.6	153	86.0
Resident on ground floor^g^	<10 years	81	76.4	134	75.3
	≥10 years	25	23.6	44	24.7
Resident in rural area where pesticides are used^g^	<10 years	88	89.8	154	93.3
	≥10 years	10	10.2	11	6.7
Passive smoking^g^	<10 years	52	49.1	95	54.3
	≥10 years	54	50.9	80	45.7
Coal/wood-burning stove^g^	<10 years	95	93.1	161	96.4
	≥10 years	7	6.9	6	3.6
Busy road <100 m^g^	<10 years	49	46.2	72	40.7
	≥10 years	57	53.8	105	59.3
Fuel storage depot <200 m^g^	<10 years	102	96.2	171	96.6
	≥10 years	4	3.8	6	3.4
Petrol station <200 m^g^	<10 years	63	59.4	122	68.9
	≥10 years	43	40.6	55	31.1
Dry cleaner <200 m^g^	<10 years	77	72.6	127	73.0
	≥10 years	29	27.4	47	27.0
Shoe/textile factory <200 m^g^	<10 years	102	96.2	170	96.6
	≥10 years	4	3.8	6	3.4
Rubbish tip <200 m^g^	<10 years	105	99.1	176	99.4
	≥10 years	1	0.9	1	0.6
Engineering industry <200 m^g^	<10 years	103	97.2	168	95.5
	≥10 years	3	2.8	8	4.5
Mine <200 m^g^	<10 years	106	100.0	177	100.0
	≥10 years	0	0.0	0	0.0
Car repair shop <200 m^g^	<10 years	90	86.5	155	88.1
	≥10 years	14	13.5	21	11.9
TV/radio transmitter <200 m^g^	<10 years	86	86.0	132	79.0
	≥10 years	14	14.0	35	21.0
Power line <200 m^g, h^	<10 years	71	73.2	117	73.6
	≥10 years	26	26.8	42	26.4
Power station <500 m^g, h^	<10 years	97	100.0	158	99.4
	≥10 years	0	0.0	1	0.6

^a^Unmarried, separated, divorced, or widow/widower

^b^At the end of time window

^c^Mother, father, sister, or brother with HM

^d^Specifically: ataxia-telangiectasia, Down syndrome, neurofibromatosis type 1, inherited immunodeficiency, MLL rearrangement, TEL/AML1 rearrangement, hepatitis A, HIV infection, HTLV-II infection, or other RNA virus infection

^e^Occam literature matrix [33]

^f^MATline matrix of carcinogenic chemicals [32]

^g^Participants with information on residential history during the time window, 106 cases and 178 matched controls

^h^Distance calculated from geo-location of residence, assessed only for cases/controls always resident in the study area during the time window

### Estimation of confounding effect of occupational exposure

For each case/control, we calculated the total number of years (not necessarily consecutive) within the time window in a given occupation. We considered that participants were occupationally exposed if they were employed for at least 10 years in industries associated with HM by the MATline job-exposure matrix or the OCCAM literature matrix (both consulted online in April 2011 [32, 33]). According to the MATline matrix, persons are occupationally exposed if employed in industries expected to use benzene, vinyl chloride, ethylene oxide, 1,3-butadiene, formaldehyde, crude coal tar, low- or high-temperature coal tar, pitch, X-rays, or gamma rays. According to the OCCAM literature matrix, participants are occupationally exposed if employed in industries associated with increased rates of HM in at least four peer-reviewed scientific articles.

### Statistical methods

Time-weighted average proximity was categorized into tertiles of the distribution in controls. Distance of main residence to refinery and distance of most recent residence to refinery were categorized as ≤3, >3 to ≤6, >6 km.

However, none of these exposure proxies took account of the time (on a daily basis) actually spent in the residences and are therefore subject to misclassification in estimating residential exposure. We therefore performed separate analyses on females and the elderly (females may more often be homemakers or unemployed than males in the area investigated) [34], and elderly persons are expected to spend most time of their time at home). Sex and age were the only information items available for all study participants. We also carried out separate analyses on participants likely to spend most of their time at home. To do this, we used information from the questionnaires to identify those who were retired, homemakers, or unemployed for at least 10 years during the time window.

Odds ratios (ORs) of HM death by tertiles of exposure proxies were estimated by conditional logistic regression. We also ran (unconditional) polynomial logistic regression model to estimate ORs of death for leukemia, non-Hodgkin lymphoma, and multiple myeloma (death for Hodgkin disease was not evaluated because only three cases were available) by tertiles of residential proximity. We estimated ORs for tertiles of increasing exposure (residential proximity) compared to lowest exposure, with two-sided 95 % confidence intervals (CIs). The likelihood ratio test was used to assess linear trend in ORs with increasing residential proximity as a three-level categorical variable. *p* values ≤0.05 were considered significant. The analyses were performed with the Stata statistical package, release 9.0 (Stata Corporation, College Station, TX, USA).

## Results

 Table 1 shows the characteristics of the 526 study participants (177 cases and 349 matched controls) and the subgroup of 295 (109 cases and matched 186 controls) whose relatives were interviewed. Most cases (44 %) had leukemia, followed by non-Hodgkin lymphoma (32 %). The sex distribution of cases was close to 50:50, while the ≥75-year age class contained proportionately more females. The proportion of participants who were long-term retired, homemakers or unemployed was lower in cases (54 %) than controls (61 %) (Table 1). However, we excluded cases from the logistic analysis for which at least one control with similar retired/homemaker/unemployed status was unavailable.

For cases/controls whose relatives were interviewed, most potential confounding variables did not differ between cases and controls. Exceptions were first degree relative to HM (not necessarily a confounder because HM in relatives might be related to exposure), smoking, residence in urban area, and residence close to petrol station (Table 2). However, when included in the logistic regression, no variable had a substantial effect on OR estimates.

Table 3 shows, for the entire set, those ≥75 years, females, and males, ORs of HM death in relation to the proximity of habitation to the refinery. No excess risk of death was found for the entire set, or for males. For those ≥75 years, risk of HM death was increased (not significant) in the second tertile (OR 1.63, 95 % CI 0.93–2.85) compared to the first, but not in the third tertile (OR 0.99, 95 % CI 0.53–1.83), with a nonsignificant *p* trend (0.842). The risk for females was significantly greater in the second tertile (OR 2.74, 95 % CI 1.48–5.09), but not in the third (OR 1.49, 95 % CI 0.76–2.92) compared to the first, with a nonsignificant *p* trend (0.184).

**Table 3:** Odds ratios (ORs), with 95 % confidence intervals (CI) for HM mortality, by time-weighted average proximity of residence to refinery for 177 cases and 349 matched controls

Residential^a^ proximity, tertiles	No. cases/No. controls	OR^b^ (95 % CI)
All study participants		
1, reference	53/117	1
2	72/115	1.37 (0.90–2.09)
3	52/117	0.97 (0.62–1.52)
* p* trend		0.970
Participants ≥75 years^c^		
1, reference	29/68	1
2	44/63	1.63 (0.93–2.85)
3	24/53	0.99 (0.53–1.83)
* p* trend		0.842
Females		
1, reference	19/67	1
2	42/48	2.74 (1.48–5.09)
3	27/58	1.49 (0.76–2.92)
* p* trend		0.184
Males		
1, reference	34/50	1
2	30/67	0.69 (0.38–1.25)
3	25/59	0.64 (0.34–1.21)
* p* trend		0.154

^a^ Residential information obtained from municipal records

^b^ORs estimated by conditional logistic regression

^c^Ninety-seven cases (36 females) and 184 matched controls (106 females)

Table 4 shows, for participants whose relatives were interviewed, ORs for any HM death, and for leukemia plus non-Hodgkin lymphoma death: Results are presented for all participants, females, and the retired-homemaker-unemployed category (both sexes) separately. For all participants and females whose relatives were interviewed, risks of HM death were comparable with those of their respective entire sets (irrespective of whether relative interviewed; see Table 3), suggesting that those with relatives interviewed did not constitute biased samples. For the retired-homemaker-unemployed category, however, risk of HM and leukemia plus non-Hodgkin lymphoma death increased with increasing proximity. For HM, ORs were 2.41 (95 % CI 0.98–5.89) for the second, and 2.19 (95 % CI 0.85–5.70) for the third tertile of proximity (*p* trend 0.079). For leukemia and non-Hodgkin lymphoma death, ORs were 3.44 (95 % CI 1.04–11.37, significant) for the second, and 3.25 (95 % CI 0.82–12.87) for the third tertiles of proximity, compared to the first (*p* trend 0.083).

**Table 4:** Odds ratios (ORs), with 95 % confidence intervals (CI) for HM mortality, by time-weighted average proximity of residences to refinery for 109 cases and 186 matched controls with relative interviewed

Residential^a^ proximity, tertiles	All haematological malignancies		Leukemia plus non-Hodgkin lymphoma	
	No. cases/No. controls	OR^b^ (95 % CI)	No. cases/No. controls	OR^b^ (95 % CI)
All participants with relative interviewed				
1, reference	36/62	1	27/47	1
2	42/68	1.08 (0.63–1.87)	34/53	1.12 (0.60–2.09)
3	31/56	0.90 (0.51–1.62)	24/46	0.86 (0.43–1.70)
* p* trend		0.770		0.688
Females with relative interviewed				
1, reference	12/38	1	9/28	1
2	24/26	2.56 (1.16–5.66)	18/19	2.55 (1.03–6.04)
3	16/25	1.52 (0.64–3.61)	11/20	1.30 (0.45–3.76)
*p* trend		0.207		0.409
Participants, relative interviewed, retired, homemaker, or unemployed^c^				
1, reference	11/32	1	6/21	1
2	26/30	2.41 (0.98–5.89)	21/23	3.44 (1.04–11.37)
3	16/17	2.19 (0.85–5.70)	11/12	3.25 (0.82–12.87)
*p* trend		0.079		0.083

^a^ Residential information obtained from municipal records

^b^ORs estimated by conditional logistic regression

^c^Retired, homemaker, or unemployed for at least 10 years during study time window: 53 cases (34 females) and 79 matched controls (53 females)

Polynomial logistic regression analysis (data not presented in tables) for males revealed no relation of proximity to any specific HM. For the entire set of females, age-adjusted ORs for leukemia death were 2.82 (95 % CI 1.12–7.15, significant) for the second, and 1.61 (95 % CI 0.61–4.26) for the third tertile compared to the first, with a nonsignificant *p* trend (0.344). ORs for non-Hodgkin lymphoma death in females were 2.58 (95 % CI 0.95–6.96) in the second and 1.35 (95 % CI 0.46–3.98) in the third tertile of proximity, with a nonsignificant *p* trend (0.553). ORs for multiple myeloma death in females were 4.54 (95 % CI 1.39–14.82, significant) in the second tertile, and 1.74 (95 % CI 0.46–6.49) in the third tertile of proximity compared to the first, again with a nonsignificant *p* trend (0.425).

## Discussion

We found that persons who probably spent most of their time at home (females, retired-homemakers-unemployed, and persons of 75 or more years) had a greater risk of HM death if their home was close to the refinery than if they lived more distant from the refinery (Tables 3, 4). For the retired-homemaker-unemployed category, the risk of leukemia plus non-Hodgkin lymphoma death increased markedly with increasing time-weighted average proximity to the plant. For other sets (all males, all participants), proximity had no influence on risk of HM death. The polynomial logistic regression analysis on all females also indicated that proximity of residence to the refinery was associated (significantly for the second tertile) with the increased risk of death from leukemia or multiple myeloma.

Our exposure proxy (time-weighted average proximity of residence to the refinery) took account of both the distance of a habitation from the refinery and the time lived in each habitation over the 15-year time window. This proxy may misclassify exposure as it does not consider daily time spent at home, the location of the workplace, or plant emissions. We tried to address time spent at home and workplace location by investigating subgroups that plausibly spent greater fractions of their daily lives at home: females (in this area of Italy, women tend to be at home more than men), and those of 75 years or older. These groups had increased risks in the second tertile of proximity (Table 3). Our interviews of the relatives of controls and cases also allowed us to identify participants who were long-term retired, homemakers or unemployed and who presumably spent much of their time at home. This provided further evidence of an effect of proximity on risk of HM death, specifically leukemia and non-Hodgkin lymphoma death. For the retired-homemaker-unemployed group, 34 % of whom were males, the misclassification of residential exposure due to the variation of daily time spent at home is likely to be lower than for other participants. However, this group might not be representative of the entire retired-homemaker-unemployed set, and differential bias cannot be ruled out due to the possibility that participants whose relatives were interviewed differed in terms of the distribution of exposure from the distribution in the entire subgroup.

We used the relative interviews as an additional source of information on residential history (the main source being municipal files). For these, there was good agreement between the two sets of residential histories (Cohen’s κ 0.810, *p* < 0.001, for tertiles of average proximity).

As regards emissions, systematic air quality monitoring of the area around the refinery began in 2005, so there are no reliable data for the time window preceding HM deaths (January 1994–December 2003). However, sporadic non-standardized measurements indicated the presence of atmospheric benzene during the study window. Distance from the plant is an acceptable proxy for exposure when air quality information is unavailable, particularly when residential history and other factors such as prevailing wind direction are also taken into account [36]. In fact, studies have used various approaches to assess exposure. One ecological study used a dispersion model based on measurements and emission data [14], and another incorporated prevailing wind direction [13] to define areas around refineries with different exposure levels. Other ecological studies considered only information related to current residence: census tract [17], or municipality [18]. Some case-control studies considered distance of main residence to plant [10, 16], or time spent in residences close to the plant [15]. Another case-control study, which found that residential petrochemical exposure was a significant risk factor for leukemia in persons of age 20–29 years, used a composite exposure proxy taking account of distance, time, and prevailing wind direction [11]. All these proxies (including the one we used) may be subject to non-differential misclassification of exposure that can only underestimate the association between death and proximity to the plant.

In ancillary analyses, we used proximity of residence lived in for the longest time and proximity of most recent residence. We found estimates of HM death risk compatible with those in our main analyses. For the retired-homemaker-unemployed category, ORs for leukemia plus non-Hodgkin death for increasing categories of proximity (>6, 3–6, ≤3 km) were 1, 2.16 (95 % CI 0.62–7.53), and 3.43 (95 % CI 0.85–13.84) with *p* trend 0.074 for residence lived in for the longest time. For the most recent residence, the ORs were 1, 3.14 (95 % CI 0.81–12.25) and 3.96 (95 % CI 0.92–17.14), *p* trend 0.068. These findings should be compared with those presented in Table 4.

One possible reason why residential proximity was significantly associated with all HM mortality in females but not males (Table 3) is that women in this part of Italy spend more time at home than men. Another possibility is that benzene may affect women more. Benzene levels have been found to be higher in women than males, in the general population (not significant) and in exposed workers (significant) for similar levels of exposure [37, 38]. This has been suggested as due to the higher percentage of body fat in women: Absorbed benzene would be released more slowly from fat to blood in women, so that they may incur greater exposure effect than males [39]. It is also noteworthy that ORs for males in the second and third tertiles were <1 (Table 3) indicating less HM deaths than expected for males living closest to the refinery. However, among long-term retired or unemployed men, the ratio of cases to controls increased with increasing tertiles of residential proximity: 4/8, 9/13, 6/5 (too few cases to analyze logistically). These considerations suggest that the fewer male HM deaths among those living close to the refinery could be due to anticipated death from other diseases linked to occupational exposure, a possibility that merits further investigation.

Several studies, using various approaches, have found that living close to a petrochemical plant was associated with increased risks of developing or dying from HMs [10, 17, 18]. Our estimates of risk of HM mortality are likely to underestimate the real health impact associated with exposure to the Falconara refinery since those who developed HMs and were still alive at study closure would not have been counted. Around the study period, population-based five-year relative survival in Italy (all ages, males and females combined) was reported as 81 % for Hodgkin disease, 56 % for non-Hodgkin lymphoma, 43 % for multiple myeloma, and 44 % for leukemia [40].

Although HMs are a heterogeneous group of diseases of diverse etiology, incidence, and prognosis, we classified them as Hodgkin disease, non-Hodgkin lymphoma, multiple myeloma, and leukemia. The main reason for adopting this crude and obsolete classification is that we analyzed death certificates, which did not usually provide more precise diagnoses, in part because HMs were similarly classified by revision nine of the International Classification of Disease [35] which was then-current during the study period.

To conclude, the present study found that the risk of HM death was unrelated to proximity for the entire set, but risk increased with proximity for females and persons who probably spent most of their time at home. Non-response bias could have either overestimated or underestimated risk findings for participants whose relatives were interviewed; however, overall our data probably underestimate the health impact of residential proximity to the refinery because we assessed risk of HM death and not risk of HM occurrence.
